# Occurrence and Antibiotic Resistance of *Vibrio parahaemolyticus* from Shellfish in Selangor, Malaysia

**DOI:** 10.3389/fmicb.2015.01417

**Published:** 2015-12-15

**Authors:** Vengadesh Letchumanan, Priyia Pusparajah, Loh Teng-Hern Tan, Wai-Fong Yin, Learn-Han Lee, Kok-Gan Chan

**Affiliations:** ^1^Division of Genetics and Molecular Biology, Institute of Biological Sciences, Faculty of Science, University of MalayaKuala Lumpur, Malaysia; ^2^Biomedical Research Laboratory, Jeffrey Cheah School of Medicine and Health Sciences, Monash University MalaysiaBandar Sunway, Malaysia

**Keywords:** *Vibrio parahaemolyticus*, shellfish, MAR index, antibiotic resistance, plasmid curing, plasmid profile

## Abstract

High consumer demand for shellfish has led to the need for large-scale, reliable shellfish supply through aquaculture or shellfish farming. However, bacterial infections which can spread rapidly among shellfish poses a major threat to this industry. Shellfish farmers therefore often resort to extensive use of antibiotics, both prophylactically and therapeutically, in order to protect their stocks. The extensive use of antibiotics in aquaculture has been postulated to represent a major contributing factor in the rising incidence of antimicrobial resistant pathogenic bacteria in shellfish. This study aimed to investigate the incidence of pathogenic *Vibrio parahaemolyticus* and determine the antibiotic resistance profile as well as to perform plasmid curing in order to determine the antibiotic resistance mediation. Based on colony morphology, all 450 samples tested were positive for *Vibrio* sp; however, *tox-R* assay showed that only 44.4% (200/450) of these were *V. parahaemolyticus*. Out of these 200 samples, 6.5% (13/200) were *trh*-positive while none were *tdh-*positive. Antibiotic resistance was determined for all *V. parahaemolyticus* identified against 14 commonly used antibiotics and the multiple antibiotic resistance index (MAR) was calculated. The isolates demonstrated high resistance to several antibiotics tested- including second and third-line antibiotics- with 88% resistant to ampicillin, 81% to amikacin,70.5% to kanamycin, 73% to cefotaxime, and 51.5% to ceftazidime. The MAR index ranged from 0.00 to 0.79 with the majority of samples having an index of 0.36 (resistant to five antibiotics). Among the 13 *trh*-positive strains, almost 70% (9/13) demonstrated resistance to 4 or more antibiotics. Plasmid profiling for all *V. parahaemolyticus* isolates revealed that 86.5% (173/200) contained plasmids - ranging from 1 to 7 plasmids with DNA band sizes ranging from 1.2 kb to greater than 10 kb. 6/13 of the pathogenic *V. pathogenic* strains contained plasmid. After plasmid curing, the plasmid containing pathogenic strains isolated in our study have chromosomally mediated ampicillin resistance while the remaining resistance phenotypes are plasmid mediated. Overall, our results indicate that while the incidence of pathogenic *V. parahaemolyticus* in shellfish in Selangor still appears to be at relatively reassuring levels, antibiotic resistance is a real concern and warrants ongoing surveillance.

## Introduction

*Vibrio parahaemolyticus* is a Gram-negative bacterium that is widely disseminated in marine and estuarine environments worldwide (Su and Liu, 2007; Ceccarelli et al., 2013; Zhang and Orth, 2013; Letchumanan et al., 2014; Velazquez-Roman et al., 2014; Wu et al., 2014). While the majority of strains isolated from environmental sources are innocuous members of marine microbiota, a small number of *V. parahaemolyticus* strains are capable of causing human illness and are often associated with food borne gastroenteritis or diarrhea (Nair et al., 2007; Hazen et al., 2015; Raghunath, 2015). The virulent strains are discerned from avirulent strains by the presence of toxigenic genes namely, thermostable direct hemolysin (*tdh*) and/or *tdh*-related (*trh*) hemolysin genes (Letchumanan et al., 2014).

Although *V. parahaemolyticus* is commonly present in seafood, most of these isolates are regarded as non-pathogenic to human (Nishibuchi and Kaper, 1995; Velazquez-Roman et al., 2012; Raghunath, 2015). The strains isolated from environmental samples usually lack the pathogenic genes thermostable direct hemolysin (*tdh*) and/or TDH-related hemolysin (*trh*) which are responsible for causing diseases in human and marine animals (Deepanjali et al., 2005; Canizalez-Roman et al., 2011; Gutierrez West et al., 2013). Previous study reported, around 0–6% of the environmental *V. parahaemolyticus* strains carry *tdh* and/or *trh* genes (DePaola et al., 2000; Vuddhakul et al., 2000; Wong et al., 2000; Alam et al., 2002; Hervio-Heath et al., 2002; Haley et al., 2014). These two genes are considered major virulence factors of *V. parahaemolyticus* (Kaysner and DePaola, 2001; Zhang and Austin, 2005; Xu et al., 2014).

There are many methods utilized for the detection of *V. parahaemolyticus*. The standard method for detection and identification of *V. parahaemolyticus* uses microbiological media enrichments such as Thiosulfate Citrate Bile Salt (TCBS) agar, Alkaline Peptone Water (APW) along with a range of biochemical tests (Vincent et al., 2015). These methods are valuable for estimation of the total load of *V. parahaemolyticus* in a sample. This then enables estimation of potential risk for presence of pathogenic strains (Malcolm et al., 2015). Routine phenotyping and biochemical identification methods of *V. parahaemolyticus* are often complicated when the strains are isolated from seafood or marine surroundings (Nishibuchi, 2006). In order to enable rapid and accurate identification of *V. parahaemolyticus*, a combination of conventional and molecular approaches using PCR assay is often adapted by researchers. At present, pathogenic *V. parahaemolyticus* isolates in seafood and environmental samples are identified using PCR-based methods that amplify *tox-R* (the toxin operon gene that is well conserved among *V. parahaemolyticus*), *tdh* and *trh* gene sequences (Panicker et al., 2004; Yamamoto et al., 2008; Paydar et al., 2013; Law et al., 2015; Malcolm et al., 2015). PCR primers can be multiplexed in a single reaction to increase the detection limit or tailored as real-time PCR assay to provide rapid results (Grant et al., 2006; Zhang et al., 2014).

An increasing population with increased purchasing power in globally has increased the demand for and export potential of seafood; resulting in steady expansion of the Asian aquaculture industry (Rico et al., 2012). However, as aquaculture practices have intensified, the sector has been constantly challenged by aquatic animal health problems which are a major constraint to the development and expansion of the aquaculture sector (Bondad-Reantaso et al., 2005). Hence, aquaculture farmers rely on a wide range of antibiotics to prevent (prophylactic use) and treat (therapeutic use) bacterial infections in fish and other invertebrates (Cabello et al., 2013). Oxytetracycline, tetracycline, quinolones, sulphonamides and trimethoprim are among the antimicrobials permitted and utilized in the Asian aquaculture industry (Rico et al., 2012; Yano et al., 2014). The extensive use of antibiotics and other chemotherapeutics in aquaculture fields has caused the emergence of antibiotic resistant strains in the environment. Every year, more and more pathogenic *Vibrio* species have been reported to develop increasing levels of resistance toward most of the clinically used antibiotics (Letchumanan et al., 2015b).

Generally, *Vibrio* species are known to be highly susceptible to most clinically used antibiotics (Mala et al., 2014; Shaw et al., 2014; Letchumanan et al., 2015a). It is noted that most of the genetic determinants that confer antibiotic resistance are located in the plasmid. Plasmids are one of the important mediators that facilitate the transfer of antibiotic resistant genes and can be transmitted to the next generation via vertical gene transfer or exchanged with other bacteria via horizontal gene transfer (Okamoto et al., 2009; Manjusha and Sarita, 2011). Plasmid profile determination represents one of the earliest DNA based assay used for epidemiological studies and remains useful today (Meyer, 1988). Previous studies have reported that *Vibrio* sp. contain plasmids and there is a correlation between possessions of plasmid with antibiotic resistance (Molina-Aja et al., 2002; Zulkifli et al., 2009). Plasmid curing of bacteria is a possible way to eliminate the plasmids which may then allow determination of the mode of antibiotic resistance mediation. Plasmid curing protocols for *Vibrio* sp. involves chemical agents such as ethidium bromide (EB), acridine orange (AO) and sodium dodecyl sulphate (SDS), and physical agents such as treatment with ultraviolet and growth at elevated temperature (Liu et al., 2012).

Shellfish mainly resides in coastal and estuarine environments. Due to the nature of their habitat, shellfish contains diverse bacterial microbiota including *Vibrio* sp. (Romalde et al., 2014). Previous studies have found high prevalence of *V. parahaemolyticus* in shellfish in Malaysia (Zulkifli et al., 2009; Al-Othrubi et al., 2014; Sahilah et al., 2014; Tang et al., 2014; Malcolm et al., 2015). The presence of potentially pathogenic *Vibrio* species in shellfish in Malaysia highlights the need for continuous monitoring as well as consumer education on food safety. Considering these factors, the present study aimed to investigate the incidence of pathogenic and multidrug resistant strains of *V. parahaemolyticus* in shellfish by both conventional and molecular methods, as well as investigating the antibiotic resistance profiles of these organisms and to attempt to determine mediations via plasmid curing.

## Materials and Methods

### Sampling

The study focused mainly on five type of shellfish; mud crab (*Scylla serrate*), flower crab (*Portunus pelagicus*), carpet clam (*Paphia textile*), hard shell clam (*Meretrix meretrix*), and mud creeper (*Cerithidea obtuse*). A total of 450 shellfish samples were purchased from three selected local wetmarkets and three local supermarkets in Selangor between January 2014 and June 2014. All the samples were sealed and transported in an ice box to the laboratory for analysis on the day of purchase.

### Enumeration and Isolation of *Vibrio* sp. in Shellfish Samples

Twenty-grams of samples (without shell) were homogenized for 60 s in a stomacher (Bagmixer 400W, Interscience, St Nom, France) with 225 mL of alkaline peptone water with 2% NaCl, pH 8.5, giving a first 10^-1^ dilution. The homogenates (1:10, 1:100, and 1:1000) were analyzed by spread-plate technique for total *Vibrio* sp. counts on Thiosulphate Citrate Bile Salt Sucrose (TCBS) agar (HiMedia, India) and incubated at 37°C for 18 h. After incubation, the total colony count and their concentrations in the original shellfish in cfu/mL was calculated.

The isolation of *V. parahaemolyticus* was carried out by incubating the homogenate at 37°C under aerobic conditions for 18 h. After incubation, a loopful of sample was streaked onto selective media, Thiosulfate Citrate Bile Salts Sucrose (TCBS) agar (HiMedia, India). Plates were then incubated at 37°C for 18 h. Characteristic green colonies (sucrose negative) on TCBS agar were considered presumptive *V. parahaemolyticus* and picked. The colonies were purified on Tryptic Soy Agar (TSA; HiMedia, India) plates supplemented with 2% w/v sodium chloride (NaCl; Vivantis, USA) and incubated at 37°C under aerobic conditions for 18 h. A loopful of pure colony was inoculated into semi-solid nutrient agar and Tryptic Soy Broth (TSB) with 30% glycerol, incubated at 37°C for 18 h and then stored until further analysis (Zarei et al., 2012; Letchumanan et al., 2015a).

### DNA Extraction

Genomic DNA of presumptive *V. parahaemolyticus* colonies was extracted using direct boiled cell lysate method (Vengadesh et al., 2012; Letchumanan et al., 2015a). In brief, *V. parahaemolyticus* colonies from semisolid nutrient agar are revived in TSB (HiMedia) with 2% w/v NaCl (Vivantis, USA) and incubated in a shaker incubator at 220 rpm for 37°C for 18 h. 1.5 mL of overnight culture suspension was subjected to centrifugation at 10,000 rpm for 5 min. The supernatant was carefully discarded, leaving the pellet dry as possible. The pellet was re-suspended in 1 mL of sterile ultrapure water, vortexed and boiled at 100°C for 7 min. The boiled cell lysate was immediately cooled on ice for 5 min before being centrifuged at 13,000 rpm for 1 min to separate the debris and DNA containing supernatant. The supernatant was carefully transferred into a new micro-centrifuge tube. The boiled cell lysate was used as the DNA template for PCR assay.

### Identification of *Vibrio parahaemolyticus* using *toxR*-Based PCR Assay

PCR amplification for detection of *V. parahaemolyticus* was performed in a final volume of 20 μL, containing 2 μL of DNA template, 10 μL of 2x *Taq PLUS* PCR Smart mix 1 (SolGent^TM^, Korea), 6 μL of sterile distilled water and 1 μL of each primer, *toxR*-F (5′-ATA CGA GTG GTT GCT GTC ATG-3′) and *toxR*-R (5′-GTC TTC TGA CGC AAT CGT TG-3′) with the expected amplicon size of 368 bp (Kim et al., 1999). The PCR amplification were performed using PCR thermocycler (Kyratec, SuperCycler Thermal Cycler, Australia) with the following cycling conditions: initial denaturation at 95°C for 4 min, 35 cycles of 94°C for 1 min, 68°C for 1 min and 72°C for 30 s, and a final elongation at 72°C for 5 min. PCR products were visualized by using 1.5% agarose gel. *V. parahaemolyticus* type strain (*V. parahaemolyticus* NBRC12711) and *V. vulnificus* type strain (*V. vulnificus* NBRC15645) was used as a positive control and negative control in every PCR reaction.

### Detection of Virulence Gene

PCR amplification for detection of *V. parahaemolyticus* virulence genes, thermostable direct haemolysin (*tdh*) and thermostable-related direct haemolysin (*trh*) was performed in duplex PCR using specific primers adapted from Bej et al. (1999). The PCR was carried out in a final volume of 20 μL, containing 2 μL of DNA template, 10 μL of 2x *Taq PLUS* PCR Smart mix 1 (SolGent^TM^, Korea), 4 μL of sterile distilled water, and 1 μL of each primer. The PCR amplifications were performed using a Thermocycler (Kyratec, SuperCycler Thermal Cycler, Australia) with the following cycling conditions: initial denaturation at 94°C for 3 min, 30 cycles of 94°C for 1 min, 58°C for 1 min and 72°C for 1 min, and a final elongation at 72°C for 5 min. All PCR products were visualized by using 1.5% agarose gel. The *V. parahaemolyticus* type strain (*V. parahaemolyticus* NBRC12711) was used as a positive control and *V. vulnificus* type strain (*V. vulnificus* NBRC15645) was used as a negative control in every PCR reaction.

### Antibiotic Susceptibility Test

Antibiotic disks (Oxoid, UK) infused with 14 antibiotics namely ampicillin (10μg), ampicillin/sulbactam (30 μg), amikacin (30 μg), cefotaxime (30 μg), ceftazidime (30 μg), chloramphenicol (30 μg), gentamicin (30 μg), imipenem (10 μg), kanamycin (30 μg), levofloxacin (5 μg), nalidixic acid (30 μg), oxytetracycline (30 μg), Sulfamethoxazole/trimethoprim (25 μg), and tetracycline (30 μg) were used in this study. The antibiotic susceptibility of *Vibrio* sp. isolates were studied using the disk diffusion method (Yano et al., 2014). The antibiotic disks were dispensed on Mueller Hilton agar (HiMedia, India) supplemented with 2% w/v NaCl (Vivantis, USA) plates with bacterial lawn. After incubation at 37°C for 18 h, the inhibition zone was measured and interpreted based on guidelines of the Clinical and Laboratory Standards Institute (CLSI) M45-A2 (Clinical Laboratory Standard Institute [CLSI], 2010).

### Plasmid Profiling

Plasmid profiling was carried out following an adaptation of the method from Devi et al. (2009). Briefly, *V. parahaemolyticus* cell were grown in TSB containing 2% w/v sodium chloride and incubated at 37°C in a shaker incubator (220 rpm) for 18 h. About 1.5 mL of the culture was transferred into a micro-centrifuge tube followed by centrifugation (10,000 rpm for 2 min at 4°C). The supernatant was removed by aspiration leaving the cell pellet as dry as possible. The pellet was resuspended in ice-cold 100 μl alkaline lysis solution I (Glucose 50 mM; Tris Cl 25 mM; EDTA 10 Mm) by vigorous vortexing followed by addition of freshly prepared 200 μL alkaline lysis solution II (NaOH 2N; SDS 2% w/v). The contents were mixed by vortexing rapidly after which 150 μl ice-cold solution III (Potassium acetate 5 M: 60 ml; Glacial acetic acid 11.5 ml; dissolved in 28.5 m sterile distilled water) was added to it. The tube was closed and gently vortexed for 10 s to disperse solution III through the viscous bacterial lysate.

Then the tubes were stored in ice for 5 min before being centrifuged at 12,000 rpm for 2 min at 4°C. An equal volume of phenol-chloroform (1:1, w/v) was added to the supernatant in a fresh tube, by vortexing. The contents in the micro-centrifuge tube were centrifuged at 8,000 rpm for 3 min at 4°C and the supernatant was transferred into a fresh tube. This was repeated with chloroform: isoamyl-alcohol (24:1, v/v) for removing the phenol. The double stranded DNA was precipitated with two volumes of ethanol at room temperature, followed by vortexing before it was allowed to stand for 5 min at room temperature. The aliquot was centrifuged at 12,000 rpm for 12 min at 4°C and the supernatant was removed by gentle aspiration. The pellet of double stranded DNA was rinsed with ethanol (1 ml, 70% v/v) at 4°C and centrifuged. The supernatant was removed leaving the pellet dry as possible. The pellet was air-dried before it was re-dissolved in 30 μl ultrapure water. Electrophoresis was performed using 1% agarose gel.

### Plasmid Curing

All the antibiotic resistant *V. parahaemolyticus* strains were subjected to a plasmid curing assay using AO. Isolates were grown in TSB supplemented with 0.2 mg/mL AO. The tubes were then incubated at 37°C for 24 h under constant agitation. After treatment with AO, the antibiotic susceptibility profile of the resistant phenotypes was examined. (Reboucas et al., 2011). *V. parahaemolyticus* VP152 and VP103 strains were used as a positive control in every plasmid curing assay (Letchumanan et al., 2015a).

### Statistical Analysis

The experimental data was analyzed by using SPSS software version 20. Statistical analysis was performed in order to determine whether there were any significant differences in the incidence of *V. parahaemolyticus* in the varying species of crustaceans studied and also to analyze the MAR index of resistant isolates using the Anova. The significance level was set at *p*-value of <0.05.

## Results

### Prevalence of *Vibrio parahaemolyticus* in shellfish

The conventional method based on the colonial appearance (green or bluish green colonies on TCBS agar) detected 450 presumptive *V. parahaemolyticus* isolates. To further confirm their identity, PCR was performed on all the presumptive *V. parahaemolyticus* isolates in order to confirm their identity by targeting species level toxin operon gene (*toxR*), 368bp. Only 44% (200/450) of the isolates were *toxR*-positive (**Table 1**). The number of *toxR*-positive isolates was similar at all sampling sites.

**Table 1:** The identification and detection of *toxR*+, *trh*+, and *tdh*+^∗^ in *Vibrio parahaemolyticus* isolates.

Sampling site	Seafood samples (*n* = 450)	Number of isolates (%)		
		
		Total	*toxR* + (%)	*trh* + (%)
Wetmarket A	Mud crab (*n* = 15)	15	8 (53)	0 (0)
	Swimming crab (*n* = 15)	15	11 (73)	0 (0)
	Hard shell clam (*n* = 15)	15	3 (20)	0 (0)
	Carpet clam (*n* = 15)	15	3 (20)	0 (0)
	Mud creeper (*n* = 15)	15	8 (53)	0 (0)
	
**Total**	*n* = 75	75	33 (44)	0 (0)

Wetmarket B	Mud crab (*n* = 15)	15	10 (67)	6 (60)
	Swimming crab (*n* = 15)	15	5 (33)	0 (0)
	Hard shell clam (*n* = 15)	15	4 (27)	3 (75)
	Carpet clam (*n* = 15)	15	5 (33)	2 (40)
	Mud creeper (*n* = 15)	15	12 (80)	2 (17)
	
**Total**	*n* = 75	75	36 (48)	13 (36)

Wetmarket C	Mud crab (*n* = 15)	15	9 (60)	0 (0)
	Swimming crab (*n* = 15)	15	8 (53)	0 (0)
	Hard shell clam (*n* = 15)	15	4 (27)	0 (0)
	Carpet clam (*n* = 15)	15	3 (20)	0 (0)
	Mud creeper (*n* = 15)	15	10 (67)	0 (0)
	
**Total**	*n* = 75	75	34 (45)	0 (0)

Supermarket A	Mud crab (*n* = 15)	15	6 (40)	0 (0)
	Swimming crab (*n* = 15)	15	6 (40)	0 (0)
	Hard shell clam (*n* = 15)	15	8 (53)	0 (0)
	Carpet clam (*n* = 15)	15	4 (27)	0 (0)
	Mud creeper (*n* = 15)	15	9 (60)	0 (0)
	
**Total**	*n* = 75	75	33 (44)	0 (0)

Supermarket B	Mud crab (*n* = 15)	15	7 (47)	0 (0)
	Swimming crab (*n* = 15)	15	9 (60)	0 (0)
	Hard shell clam (*n* = 15)	15	3 (20)	0 (0)
	Carpet clam (*n* = 15)	15	3 (20)	0 (0)
	Mud creeper (*n* = 15)	15	9 (60)	0 (0)
	
**Total**	*n* = 75	75	31 (41)	0 (0)

Supermarket C	Mud crab (*n* = 15)	15	6 (40)	0 (0)
	Swimming crab (*n* = 15)	15	8 (53)	0 (0)
	Hard shell clam (*n* = 15)	15	7 (47)	0 (0)
	Carpet clam (*n* = 15)	15	4 (27)	0 (0)
	Mud creeper (*n* = 15)	15	8 (53)	0 (0)
	
**Total**	*n* = 75	75	33 (44)	0 (0)



*^∗^All the *V. parahaemolyticus* isolates were negative for *tdh* gene.*

The density of total *Vibrio* count in the shellfish samples are shown in **Table 2**. The shellfish samples analyzed had a mean total *Vibrio* count range of 2.45–6.63 log cfu/g. A bacterial load of 5–7 log cfu/g is generally considered to be the level necessary to cause clinically significant infection in humans. Out of the 30 categories, 50% (15/30) were found to be >5 log cfu/g; however, this is with reference to *Vibrio* sp, not specifically *V. parahaemolyticus*. Of these 15 samples, eight were from wetmarkets and seven were from supermarkets. Mudcrab from all the locations sampled exceeded 5 log cfu/g. Carpet clams from the three wet markets all exceeded 6 log cfu/g while only one sample from the supermarket exceeded 5 log cfu/g. This pattern was reversed in mud creepers, however, with the supermarket samples showing higher *Vibrio* density. Overall, swimming crab showed the lowest density of *Vibrio* sp. density with four of the six sites sampled having mean densities of less than 3 log cfu/g.

**Table 2:** The mean of total *Vibrio* counts (log CFU/g) of each shellfish samples from respective sampling site.

Samples	Total *Vibrio* density (log CFU/g)					
	
	Wetmarket A	Wetmarket B	Wetmarket C	Supermarket A	Supermarket B	Supermarket C
Mud crab	5.53 ± 0.03	5.44 ± 0.12	5.39 ± 0.22	5.00 ± 0.20	5.33 ± 0.05	5.17 ± 0.48
Swimming crab	2.45 ± 0.21	4.52 ± 0.49	3.40 ± 0.40	2.29 ± 0.16	2.88 ± 1.24	2.59 ± 0.30
Hard shell clam	5.00 ± 0.69	4.90 ± 0.06	4.95 ± 0.13	4.13 ± 1.12	4.06 ± 0.12	4.00 ± 0.04
Carpet clam	6.63 ± 0.19	6.42 ± 0.04	6.50 ± 0.25	5.56 ± 0.47	3.03 ± 0.40	4.30 ± 0.43
Mud creeper	4.54 ± 0.25	5.23 ± 0.28	4.89 ± 0.55	5.86 ± 0.43	5.92 ± 0.19	5.90 ± 0.22
Total average of *Vibrio* count (log CFU/g)	4.83 ± 1.52^a^	5.26 ± 0.72^a^	5.03 ± 1.12^a^	4.57 ± 1.43^a^	4.24 ± 1.35^a^	4.40 ± 1.25^a^


*Values = mean ± standard deviation (*n* = 15). ^a^ Means with different superscripts in the same row indicate significant difference (*p* < 0.05).*

### Prevalence of Thermostable Direct Hemolysin *(tdh)* and TDH-Related Hemolysin *(trh)* Positive *Vibrio parahaemolyticus* Isolates

To detect pathogenic isolates, *tdh* and *trh* genes were amplified using PCR-based assay. The results of this analysis are summarized in **Table 3**. 13/200 (6.5%) *trh*-positive isolates were identified – all 13 were from shellfish samples collected from wetmarket B. Of these, six samples were mud crab, two samples were carpet clam, two samples were mud creeper and three samples were hard shell clam (**Table 1**). No *tdh*-positive *V. parahaemolyticus* isolates were detected.

**Table 3:** List of *trh-*positive *Vibrio parahaemolyticus* isolates.

Strains	Samples	Location	*toxR*-positive	*trh*-positive	Before plasmid curing			After plasmid curing	
						
					Antibiotic resistance pattern	No. of plasmid	Plasmid size	Antibiotic resistance pattern	No. of plasmids
SVP55	Mud crab	Wetmarket B	+	+	amp/ak/caz/ctx/k	None		amp/ak/caz/ctx/k	
SVP56	Mud crab	Wetmarket B	+	+	amp/ak/caz/ctx/k	None		amp/ak/caz/ctx/k	
SVP60	Mud crab	Wetmarket B	+	+	amp	None		amp	
SVP61	Mud crab	Wetmarket B	+	+	amp/ak/caz/ctx	7	1.2 kb, 1.75 kb, 3 kb, 3.1 kb, 4 kb, two above 10 kb	amp	Lost
SVP64	Mud crab	Wetmarket B	+	+	amp/ak/ctx	None		amp/ak/ctx	
SVP66	Mud crab	Wetmarket B	+	+	amp/ak/caz/ctx	None		amp/ak/caz/ctx	
SVP52	Carpet clam	Wetmarket B	+	+	amp/ak/ctx	None		amp/ak/ctx	
SVP54	Carpet clam	Wetmarket B	+	+	amp/ak/caz/ctx/k/lev	1	above 10 kb	amp	Lost
SVP73	Mud creeper	Wetmarket B	+	+	ak/ctx/k	1	above 10 kb	All susceptible	Lost
SVP75	Mud creeper	Wetmarket B	+	+	amp/ak/ctx/k	1	above 10 kb	amp	Lost
SVP69	Hard shell clam	Wetmarket B	+	+	amp/ak/caz/ctx	1	1.75 kb	amp	Lost
SVP70	Hard shell clam	Wetmarket B	+	+	amp/ak/caz/ctx/k	None		amp/ak/caz/ctx/k	
SVP72	Hard shell clam	Wetmarket B	+	+	amp/ak/caz/ctx	2	2.5 kb, one above 10 kb	amp	Lost


### Antimicrobial Susceptibilities of *Vibrio parahaemolyticus* Isolates

*Vibrio parahaemolyticus* isolates from shellfish samples were further tested for antimicrobial susceptibilities. All 14 antibiotics used in this study are among the antibiotics recommended by Centre for Disease Control and Prevention (CDC) for the treatment of *Vibrio* sp. infections that include fluoroquinolones (levofloxacin), cephalosporins (cefotaxime and ceftazidime), aminoglycosides (amikacin and gentamicin) and folate pathway inhibitors (trimethoprim-sulfamethoxazole; Daniels et al., 2000; Shaw et al., 2014). The results of this test are summarized in **Table 4**. With regard to CDC recommended antimicrobial agents, the isolates exhibited resistance to ampicillin (88.0%), amikacin (81.0%), and kanamycin (70.5%). Within the third generation cephalosporin, isolates exhibited resistance to cefotaxime (73%) and ceftazidime (51.5%). Reassuringly, the isolates tested were still susceptible to several antibiotics tested, imipenem (90.0%), chloramphenicol (88.0%), tetracycline (84.0%), ampicillin/sulbactam (67.0%), levofloxacin (61.5%) and trimethoprim-sulfamethoxazole (50%). With reference to the pathogenic strains, all the thirteen *trh*-positive isolates were resistant to ampicillin, 12/13 isolates expressed resistance to amikacin and cefotaxime, and 8/13 isolates were resistant to ceftazidime.

**Table 4:** The antibiotic resistant profile of *V. parahaemolyticus* isolates (*n* = 200).

Class of antibiotics	Antibiotics	Concentration (μg)	No. of resistant isolates (%)	No. of intermediate isolates (%)	No. of susceptible isolates (%)
Penicillins	Ampicillin	10	176 (88.0)	10 (5.0)	14 (7.0)
	Ampicillin-sulbactum	30	38 (19.0)	28 (14.0)	134 (67.0)
Cephalosporins	Cefotaxime	30	146 (73.0)	21 (10.5)	33 (16.5)
	Ceftazidime	30	103 (51.5)	46 (23.1)	51 (25.5)
Carbapenems	Imipenem	10	1 (0.5)	18 (9.0)	181 (90.0)
Aminoglycosides	Amikacin	30	162 (81.0)	25 (12.5)	13 (6.5)
	Gentamicin	30	7 (3.5)	79 (39.5)	114 (57.0)
	Kanamycin	30	141 (70.5)	54 (27.0)	5 (2.5)
Tetracycline	Tetracycline	30	26 (13.0)	6 (3.0)	168 (84.0)
	Oxytetracycline	30	32 (16.0)	99 (49.5)	69 (34.5)
Quinolones	Nalidixic acid	30	3 (1.9)	60 (37.5)	97 (60.6)
	Levofloxacin	5	14 (8.8)	47 (29.4)	99 (61.9)
Folate pathway inhibitor	Trimethoprim-sulfamethoxazole	25	11 (6.9)	69 (43.1)	80 (50.0)
Phenicols	Chloramphenicol	30	14 (8.8)	5 (3.1)	141 (88.1)


Eighty-five percentage of the isolates had multiple antibiotic resistance (MAR) index more than 0.2. Gwendelynne et al. (2005) stated that MAR indices higher that 0.2 could be a marker of contamination from high risk sources, thus indicating a potential human health risk. MAR index in this study ranged from 0.00 to 0.79, with the highest MAR index (0.79) found in a non-pathogenic isolate (SVP129) of carpet clam from supermarket B which expressed resistance to 11/14 antibiotic tested. The majority of *V. parahaemolyticus* isolates studied showed a MAR index of 0.36 (resistant to five antibiotics tested). 15% of the isolates had MAR index of 0.00–0.07, indicating the isolates were resistant to none or at least one type of the antibiotic tested.

### Plasmid Profiling and Plasmid Curing of *Vibrio parahaemolyticus* Isolates

All the *V. parahaemolyticus* isolates were tested for the presence of plasmid before curing. Plasmid profiles of the 200 *V. parahaemolyticus* isolates revealed that 173/200 isolates contained one to seven plasmids ranging from 1.2 kb to above 10 kb in size while the remaining 27 isolates did not have any plasmids. 6/13 *trh*-positive *V. parahaemolyticus* isolates (**Table 3**) contained plasmids, with all six containing at least one plasmid above 10 kb. (SVP61); *trh*-positive isolate) expressed seven plasmids with sizes of 1.2, 1.75, 3, 3.1, 4, and 2 kb above 10 kb size. SVP61 was resistant to 4/14 antibiotics tested. The study revealed that (SVP129; non-pathogenic isolate) of carpet clam from supermarket B has one plasmid above 10 kb size and is resistant to 11/14 antibiotics tested.

Plasmid curing is a method which potentially enables the elimination of plasmids from the organisms being studied which can then allow the determination of antibiotic resistance mediation by retesting the organisms after plasmid curing. Antibiotic resistance of all *V. parahaemolyticus* isolates which had underdone plasmid curing was tested by antibiotic disk diffusion method. The resistance exhibited by isolates to ampicillin and tetracycline did not vary even after curing of plasmids. With reference to the 13 *trh*-positive strains, the antibiotic resistance profile of the six plasmid containing strains altered after curing while the remaining seven were unchanged. All six were ampicillin resistant initially, and after curing, one strain became susceptible to all antibiotics tested while five of the strains (SVP61, SVP54, SVP75, SVP69, VP72) remained resistant to ampicillin and became susceptible to the other antibiotics tested. This suggests that while antibiotic resistance is mediated by both plasmid and chromosomes in pathogenic *V. parahaemolyticus* isolates, in plasmid containing strains, aside from ampicillin resistance, most of the remaining resistance phenotypes are plasmid mediated.

SVP129 isolate contained one plasmid profile with size more than 10 kn and expressed resistance to 11/14 antibiotics tested. After plasmid curing, SVP129 isolate lost its plasmid and changed its antibiotic resistance phenotype. SVP129 isolate remained resistant to 5/14 antibiotic tested namely ampicillin, oxytetracycline, chloramphenicol, tetracycline, and sulfamethoxazole/trimethoprim. The isolate showed intermediate resistance to amikacin, ceftazidime, cefotaxime, and kanamycin, while it was susceptible to gentamycin and ampicillin/sulbactam after plasmid curing assay.

## Discussion

The presence of pathogenic strains of *V. parahaemolyticus* in the shellfish we studied does raise concern as these organisms are known to be a frequent cause of food-borne gastroenteritis in humans. However, while conventional colony morphology found all samples to be contaminated with *Vibrio* sp. only 44% (200/450) of these were confirmed to be *V. parahaemolyticus* based on *tox-R* assay; and only 6.5% (13/200) of these were pathogenic strains (*trh*-positive). Conventional bacteriological methods have demonstrated a highly variable occurrence of *V. parahaemolyticus* in seafood around the world. Our results are in close agreement with those reported by Zhao et al. (2011) in China, who isolated *V. parahaemolyticus* from 59.7% shellfish samples. Our study samples exhibited a mean total *Vibrio* count range of 2.45–6.63 log cfu/g. The mean total *Vibrio* count of the shellfish samples from wetmarkets were higher compared to shellfish samples from supermarkets. However, the results obtained from one-way analysis of variance (**Table 2**), showed there were no significant difference (*p* > 0.05) between the total *Vibrio* counts of the shellfish samples from all the sampling sites. The difference in the incidence of *V. parahaemolyticus* among samples from both sampling sites could possibly be contributed by the original geographic source from which the shellfish were collected, post-harvest practices and hygiene standards applied during handling, transportation and storage of seafood products. A study in Cochin, India, reported higher *Vibrio* sp. contamination in shellfish from roadside stalls compared to markets (Sudha et al., 2014). Other studies have reported lack of hygiene, improper handling, cross contamination and difference in storage temperature as the possible cause of variation in *V. parahaemolyticus* incidences in samples from supermarket (Yang et al., 2008; Tunung et al., 2010; Sudha et al., 2014).

In order to assess the actual risk to human health posed by the presence of *V. parahaemolyticus* in seafood, incidence of pathogenic strains need to be determined via detection of the toxigenic genes responsible for causing disease in humans. Of the 200 isolates tested in this study, only 13 (6.5%) were *trh*-positive and there were no *tdh*-positive strains. This incidence is similar to the results demonstrated in Malaysia by Paydar et al. (2013) who detected 6/50 (12%) *trh*-positive *V. parahaemolyticus* among the food samples tested. In another study, a much higher incidence of *tdh*-positive and *trh*-positive in shrimp and cockles samples was reported in Malaysia with twenty six isolates were *trh*-positive and eight *tdh*-positive (Al-Othrubi et al., 2014). Normally, only 1–2 % of the environmental strains harbor the *tdh* and *trh* genes (Wong et al., 2000; Alam et al., 2002; Hervio-Heath et al., 2002; Velazquez-Roman et al., 2012; Haley et al., 2014). Studies in the USA and in Japan reported that only 3% of *V. parahaemolyticus* strains isolated from seafood and environment samples were pathogenic (DePaola et al., 2003; Mahmoud et al., 2006; Abd-Elghany and Sallam, 2013). It is also reported that environmental factors including interaction with different hosts plays an important impact in the evolution of specific pathogens (Wilson and Salyers, 2003).

The continuous and extensive use of antibiotics in the aquaculture industry favors the development of a variety of resistant isolates and the dissemination of resistance genes within the bacterial population in the environment which reflects the pattern of drug use (Tendencia and Pena, 2002; Reboucas et al., 2011). All the antibiotics tested in this study are recommended antimicrobial agents used in the treatment of *Vibrio* sp. infections, including the tetracycline, levofloxacin, cefotaxime, ceftazidime, amikacin, gentamicin, and trimethoprim-sulfamethoxazole (Daniels et al., 2000; Shaw et al., 2014). Some of these antibiotics are widely used in aquaculture industry as antimicrobial agents including oxytetracycline and chloramphenicol (Dang et al., 2007). In this study, 88% of the *V. parahaemolyticus* isolates from shellfish exhibited resistance to ampicillin. Our results are in close agreement with other studies that reported resistance to ampicillin among the *V. parahaemolyticus* isolates from seafood samples (Okuda et al., 1997; Han et al., 2007; Al-Othrubi et al., 2014; Letchumanan et al., 2015a). First generation antibiotics including ampicillin are extensively used in the aquaculture thus reducing to susceptibility and resulting in low efficacy of ampicillin for *Vibrio* sp. treatment (Sudha et al., 2014).

Resistance to third generation cephalosporin, was observed in our isolates of *V. parahaemolyticus*, with 73% resistant to cefotaxime and 51.5% resistant to ceftazidime. This is slightly less compared to 80% in a study by Jun et al. (2012) studying the resistance to third generation cephalosporin among *V. parahaemolyticus* isolates from Korean seafood. Our study is in agreement with Sahilah et al. (2014) in Terengganu, Malaysia, who reported presence of *V. parahaemolyticus* isolates from shellfish to be resistant to ceftazidime and cefuroxime. However, a study by Shaw et al. (2014) reported low percentage of *V. parahaemolyticus* from USA to be resistant to cefotaxime. The discrepancies in the literature regarding the resistance phenotype of *V. parahaemolyticus* to third generation cephalosporin could possibly due to difference in test methodology or geographical variation. In the present study, high susceptibility to imipenem (90%), chloramphenicol (88.1%) and tetracycline (84%) was observed among the *V. parahaemolyticus* isolates, much in agreement with other publications (Han et al., 2007; Sahilah et al., 2014; Sudha et al., 2014).

High MAR indices were detected in this study, ranging from 0.00 to 0.79, with 85% of the isolates having a MAR index value more than 0.2. MAR indices higher than 0.2 are markers of high risk sources, which may represent a potential human health risk (Gwendelynne et al., 2005). The most frequent MAR index of the resistant *V. parahaemolyticus* isolates tested was 0.36, indicating that the strains were resistant toward five different antibiotics. The MAR index of *V. parahaemolyticus* isolates in present study varied significantly (ANOVA, *p* < 0.05) between wetmarket and supermarket. Our results are in agreement with a study by Elexson et al. (2014) in Malaysia which reported 97.2% of *V. parahaemolyticus* isolates had MAR index more than 0.2, however, a lower percentage (14.5%) with MAR index more than 0.2 was reported by Tang et al. (2014). The huge variation in the MAR index of the *V. parahaemolyticus* isolates observed in present and past studies in Malaysia may be influenced by the variance in the resistance levels depending on the source of sample collection (Khan et al., 2007; Tunung et al., 2012). Lesley et al. (2011) suggested that the difference in geographical locations may have differential selective pressures for the antibiotic resistance level.

Among the 200 *V. parahaemolyticus* isolates, only 173 isolates (86.5%) harbored between one to seven plasmid DNA bands, which range in size from 1.2 kb to above 10 kb and the rest did not exhibit the presence of plasmid DNA. The study findings correlates with results of previous studies, which have reported that *V. parahaemolyticus* harbored plasmids (Kagiko et al., 2001; Kaufman et al., 2002; Molina-Aja et al., 2002; Manjusha et al., 2005; Zulkifli et al., 2009). The isolate (SVP129) had only one plasmid above 10 kb band size and showed resistance to 11/14 antibiotics tested. With reference to *trh*-positive strains, seven of the isolates did not have any plasmid but were resistant to more than one antibiotic tested.

When submitted to plasmid curing, (SVP129) isolate lost its plasmid and altered its resistance phenotype to the antibiotics tested. The isolate remained resistant to sulfamethoxazole/trimethoprim, ampicillin, oxytetracycline, chloramphenicol, and tetracycline. SVP129 isolate showed intermediate resistant to amikacin, ceftazidime, cefotaxime, and kanamycin, while it was susceptible to gentamicin and ampicillin/sulbactam after plasmid curing assay. AO acts as an intercalating agent that inhibits plasmid replications (Letchumanan et al., 2015b). The resistance phenotype exhibited by SVP129 could be chromosomal mediated. Meanwhile, 6/13 *trh*-positive isolates lost their plasmids after curing assay and altered their resistance phenotype to amikacin, ceftazidime, cefotaxime, kanamycin, and levofloxacin (**Table 2**).

All the ampicillin resistant isolates (176/200) and tetracycline resistant isolates (26/200) remain resistant to the respective antibiotics after plasmid curing. This suggests that the resistance phenotype to ampicillin and tetracycline expressed by the isolates could be chromosomally mediated. All the ampicillin/sulbactam resistant strains lost their plasmid after the curing assay and subsequently were susceptible to ampicillin/sulbactam suggesting that the resistance was plasmid mediated. Our results are closely in agreement with other studies that reported *Vibrio* sp., strains lost their plasmids when treated with concentration of 0.2 mg/ml AO and the isolates demonstrated changes in their resistance profile (Molina-Aja et al., 2002; Barman et al., 2010; Reboucas et al., 2011; Carvalho et al., 2013; Costa et al., 2015). A study by Reboucas et al. (2011) in Brazil, reported AO was successfully used to cure multi-resistant *Vibrio* isolates from marine shrimp and concluded the ampicillin resistance strains in study are plasmid mediated. In contrast, another study reported their isolates resistance was chromosomal mediated after AO curing treatment (Costa et al., 2014). The loss of phenotype in these studies suggest that AO produce an immediate and complete inhibition of plasmid replication, thus able to act as reliable plasmid curing agent.

Plasmid curing assay may be used to eliminate bacterial plasmids and determine antibiotic resistance mediation. The assay provides vital information which would be beneficial in the global surveillance management of environmental multidrug resistance. Reducing and improving the use of antibiotics in the aquatic environment can reduce resistance and allow the antibiotic to resurface eventually as an effective therapy (Barbosa and Levy, 2000). The management of antibiotic resistance may vary depending on the resistance location, plasmidial, or chromosomal mediated resistance. Plasmidial mediated resistance can be controlled by alternating the antibiotics used in the environments. The aquaculture industry could adapt the method of switching antibiotics used in the aquatic field from time to time in order to allow withdrawal of antibiotic resistance profile in strains (Letchumanan et al., 2015b). However, it would be a challenge to control chromosomal mediated resistance because the resistant genes reside in the chromosomes of the bacteria. Hence, non-antibiotic management strategies using bacteriophages may help to control chromosomal mediated resistance. Bacteriophages are found in abundance in the aquatic environment and may play a vital role in controlling microbial populations. Many studies have demonstrated the potential of bacteriophages to control bacterial diseases and have been shown to be effective in reducing pathogen levels in aquaculture species, such as shrimp (Karunasagar et al., 2007) and finfish (Park and Nakai, 2003).

Quorum sensing and quorum quenching may be used as an alternative method to manage antibiotic resistance. This strategy has been widely proposed to control infections in aquaculture environments by detecting bacterial cell-to-cell communication thru small signal molecules of quorum sensing (Defoirdt et al., 2004). Quorum sensing is a mechanism of gene regulation in which bacteria coordinate the expression of certain genes in response to the presence or absence of small signal molecules. The bacterial communication is then stopped through quorum quenching and hence the expression of genes is disrupted (Defoirdt et al., 2008). Hence, this knowledge and information gathered would be useful to manage the emergence of antibiotic resistance.

In summary, this study showed that only 2.8% (13/450) of the seafood samples studied contained pathogenic strains of *V. parahaemolyticus*. These 13 strains were deemed pathogenic based on *trh*-positive status. These pathogenic strains all exhibited antibiotic resistance, with almost 70% (9/13) demonstrating resistance to 4 or more antibiotics tested. Out of the 200 *V. parahaemolyticus* isolates analyzed, there was a high level of resistance to ampicillin and tetracycline as well as to some third generation cephalosporin. While the overall incidence of pathogenic strains of *V. parahaemolyticus* in Selangor is relatively reassuring, the high MAR of all the *V.parahaemolytics* tested – including the pathogenic strains- is a definite cause for concern and warrants ongoing surveillance.

## Conflict of Interest Statement

The authors declare that the research was conducted in the absence of any commercial or financial relationships that could be construed as a potential conflict of interest.
